# Women in Japanese cardiovascular surgery: 21-year trends (2004–2025), current status and future perspectives

**DOI:** 10.1007/s11748-026-02306-7

**Published:** 2026-04-30

**Authors:** Osamu Ishida, Shotaro Yoshida, Koji Tsutsumi

**Affiliations:** Department of Cardiovascular Surgery, National Defense Medical College, 3-2 Namiki, Tokorozawa-shi, Saitama, 359-8513 Japan

**Keywords:** Cardiovascular surgery, Woman in surgery, Gender gap, Workforce trend, Japan

## Abstract

**Background:**

Despite the increasing number of female medical graduates in Japan, women remain underrepresented in cardiovascular surgery.

**Methods:**

Using complete registry data from the Japanese Board of Cardiovascular Surgery (2004–2025), we examined annual proportions of female board-certified cardiovascular surgeons and newly certified surgeons. Linear regression was performed to assess trends and extrapolate future proportions. International benchmarking utilized published data from the United States, Europe, and global surveys.

**Results:**

The proportion of female board-certified cardiovascular surgeons increased from 0.7% in 2004 to 4.8% in 2025 (annual increase 0.202 percentage points; 95% CI 0.18–0.23; R^2^ = 0.973; *P* < 0.001). Among newly certified surgeons, the proportion was more variable, reaching 9.0% in 2025. Linear extrapolation projected the overall proportion to reach 5.6% in 2030 (95% prediction interval 5.0–6.1%) and 7.6% in 2040 (95% prediction interval 7.0–8.2%). The current Japanese proportion of 4.8% is approximately half the global average of 8.0% (from the 2024 EACTS global workforce analysis based on CTSNet data across 146 countries) and 42–47% of the United States (11.5%) and Europe (10.3%) in the same report.

**Conclusions:**

Over two decades, female representation in Japanese cardiovascular surgery has risen steadily to 4.8%, driven by increasing female new certifications. Japan's current level corresponds to Western proportions from the mid-2000s, reflecting a persistent international disparity. The recent physician work-style reform and ongoing efforts to improve retention may influence future trends, but longitudinal data are needed to evaluate their impact.

**Supplementary Information:**

The online version contains supplementary material available at https://doi.org/10.1007/s11748-026-02306-7.

## Introduction

The under-representation of women in cardiovascular surgery is a persistent global challenge, with marked regional variation in the pace of progress. Recent international data indicate that women comprise only 8.0% of the worldwide cardiac surgical workforce, ranging from 11.4% in North America and 10.3% in Europe to considerably lower proportions in other regions [1]. This study was motivated by the strikingly low female proportion of 2.9% reported for East Asia in the same report, which appeared discrepant with our clinical observations in Japan. We therefore sought to provide a detailed, longitudinal analysis using complete official registry data to objectively characterize the trend in Japanese cardiovascular surgery and to facilitate accurate international comparison. In Japan, although female physicians accounted for 23.6% of the total workforce in 2022 [2], and the proportion of female entrants to national university medical schools reached 38.2% in 2025 [3], cardiovascular surgery remains one of the least gender-diverse specialties.

Historical trends in Western countries demonstrate more rapid advancement. In the United States and Europe, female representation in cardiothoracic surgery has risen substantially over the past two decades, supported by structured mentorship programs and dedicated scholarships programs [4, 5].

In contrast, the specialty in Japan has long been characterized by very low female participation. Barriers such as prolonged operative hours and higher attrition rates among female surgeons, often linked to childbirth and child-rearing responsibilities, have been reported in surgical fields [1, 6]. The physician work-style reform introduced in 2024, which limits overtime hours, has been expected to improve retention among female surgeons by alleviating long working hours and on-call burdens. However, specialty-specific data on attrition rates in cardiovascular surgery are currently unavailable, and future longitudinal studies are needed to evaluate its effectiveness.

This study analyzes the 21-year trend from 2004 to 2025 in the proportion of female board-certified cardiovascular surgeons in Japan using complete registry data from the Japanese Board of Cardiovascular Surgery. It describes the annual proportions overall and among new certifications, models long-term trends with linear regression, and benchmarks the current Japanese proportion against contemporary international data to assess the extent of the gender disparity.

## Methods

### Study design and data sources

This retrospective longitudinal study utilized complete official data provided directly by the Japanese Board of Cardiovascular Surgery for all board-certified cardiovascular surgeons in Japan from 2004 to March 2025. The registry contains the annual census of certified surgeons, including year of first certification and sex (determined from official name lists). The registry data are available only from 2004 onward, as this corresponds to the year when the current board certification system for cardiovascular surgery was established in Japan. While earlier certification practices existed prior to 2004, those data are not included in the current registry maintained by the Japanese Board of Cardiovascular Surgery and were not available for this study.

### Variables

The primary outcome was the annual proportion of female board-certified cardiovascular surgeons, calculated as (number of female surgeons ÷ total number of active certified surgeons) × 100. Secondary outcomes included the absolute number of female surgeons, the proportion of females among newly certified surgeons each year.

### Statistical analysis

Simple linear regression was performed with calendar year as the independent variable and female proportion (%) as the dependent variable. The slope represents the average annual increase in percentage points, with 95% prediction intervals calculated. Future projections (2030, 2035, 2040) were derived from the fitted model. Prediction intervals were computed using RStudio (R version 4.5.2, The R Foundation for Statistical Computing).

Net attrition was estimated using a balance equation based on the total number of certified surgeons and the number of new certifications. For fiscal year *t*, the net number of leavers was calculated as *L*_*t*_ = *N*_*t−1*_ + New_*t*_ − *N*_*t*_, where *N*_*t−1*_ and *N*_*t*_ denote the total number of certified surgeons in the previous and current years, respectively, and New_*t*_ denotes new certifications in year *t*. The annual attrition rate (%) was calculated as [*N*_*t−1*_ + New_*t*_ − *N*_*t*_ / *N*_*t−1*_] × 100. When the estimated net attrition was zero or negative (i.e., no net loss or a net gain, reflecting inflows not captured by new certifications), the attrition rate was reported as 0.0%. This calculation captures the net change in the certified workforce, which includes all reasons for removal from the registry (e.g., non-renewal of certification, voluntary withdrawal from clinical activity, retirement, death, or transfer to another specialty). Therefore, the estimates represent net loss from the certified pool rather than exclusively withdrawal from clinical activity. To summarise attrition over a multi-year period (years *t* = *a*,…,*b*), we calculated a weighted mean net attrition rate as ${\overline{\mathrm{r}}} = {1}00 \times \sum\nolimits_{t = a}^{b} {L_{t} } /\sum\nolimits_{t = a}^{b} {N_{t - 1} }$, where *L*_*t*_ is the estimated net attrition count for year *t* defined above. These calculations were performed separately for women and men; male counts were derived as the total minus female counts. Period estimates were reported separately for women and men.

To assess the extent of the gender disparity, international benchmarking was performed using data from the 2024 EACTS global workforce analysis and published reports from the Society of Thoracic Surgeons (STS), which includes cardiothoracic surgery with cardiac surgery as a major component in the United States.

All analyses and graph preparation were performed using GraphPad Prism (Version 7.04, GraphPad Software, San Diego, CA) and RStudio (R version 4.3.2). Two-sided *P*-values < 0.05 were considered statistically significant.

### Ethical considerations

The study used fully anonymized, aggregated data officially provided by the Japanese Board of Cardiovascular Surgery for research purposes. No individual identifiable information was included. The study was conducted in accordance with the Declaration of Helsinki.

## Results

### Overall trends in female representation

**Fig. 1:**
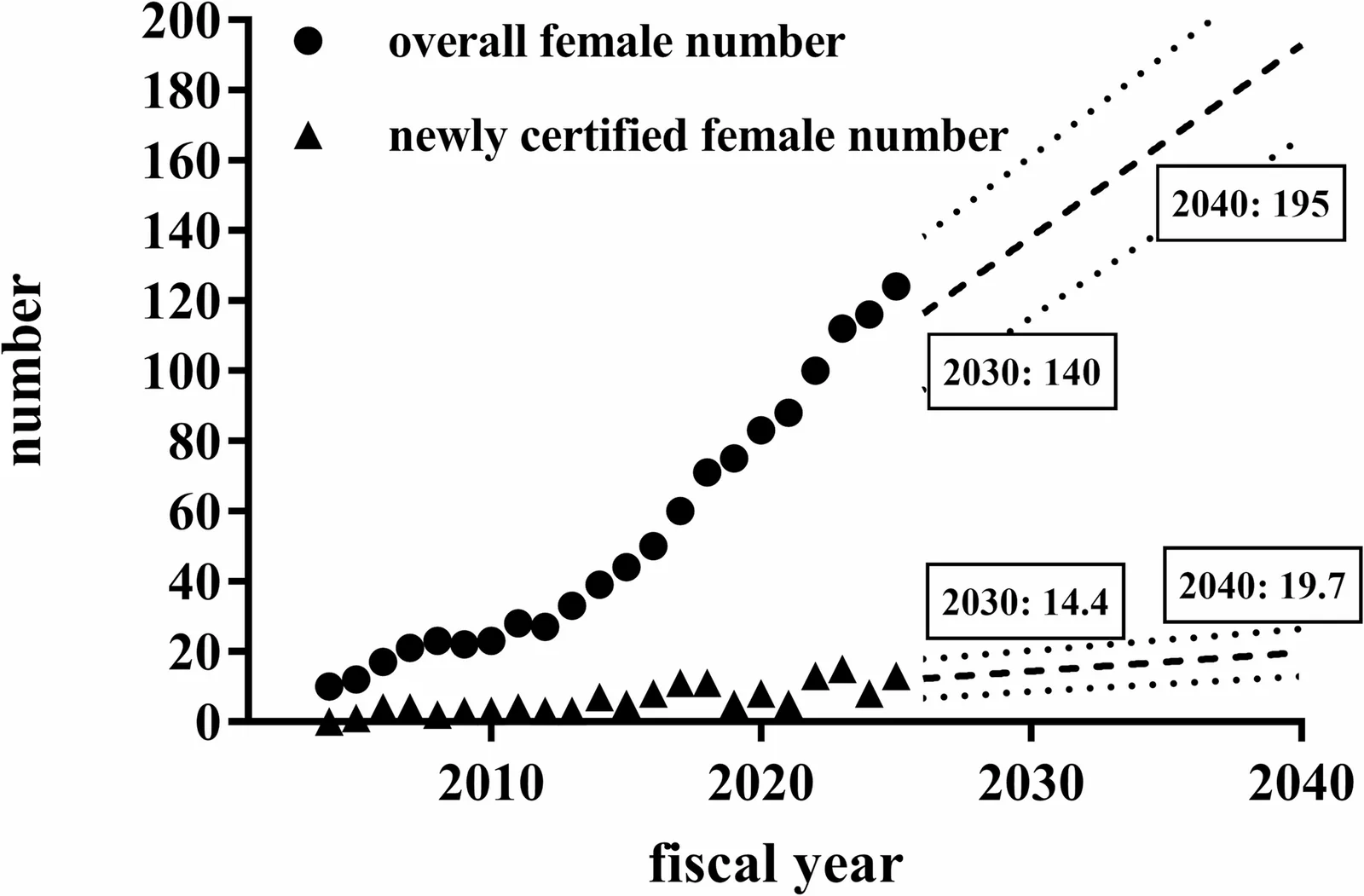
Number of Board-Certified Cardiovascular Surgeons in Japan (Female and New Female Certifications), 2004–2025, with Linear Regression and Prediction Intervals Extended to 2040

**Fig. 2:**
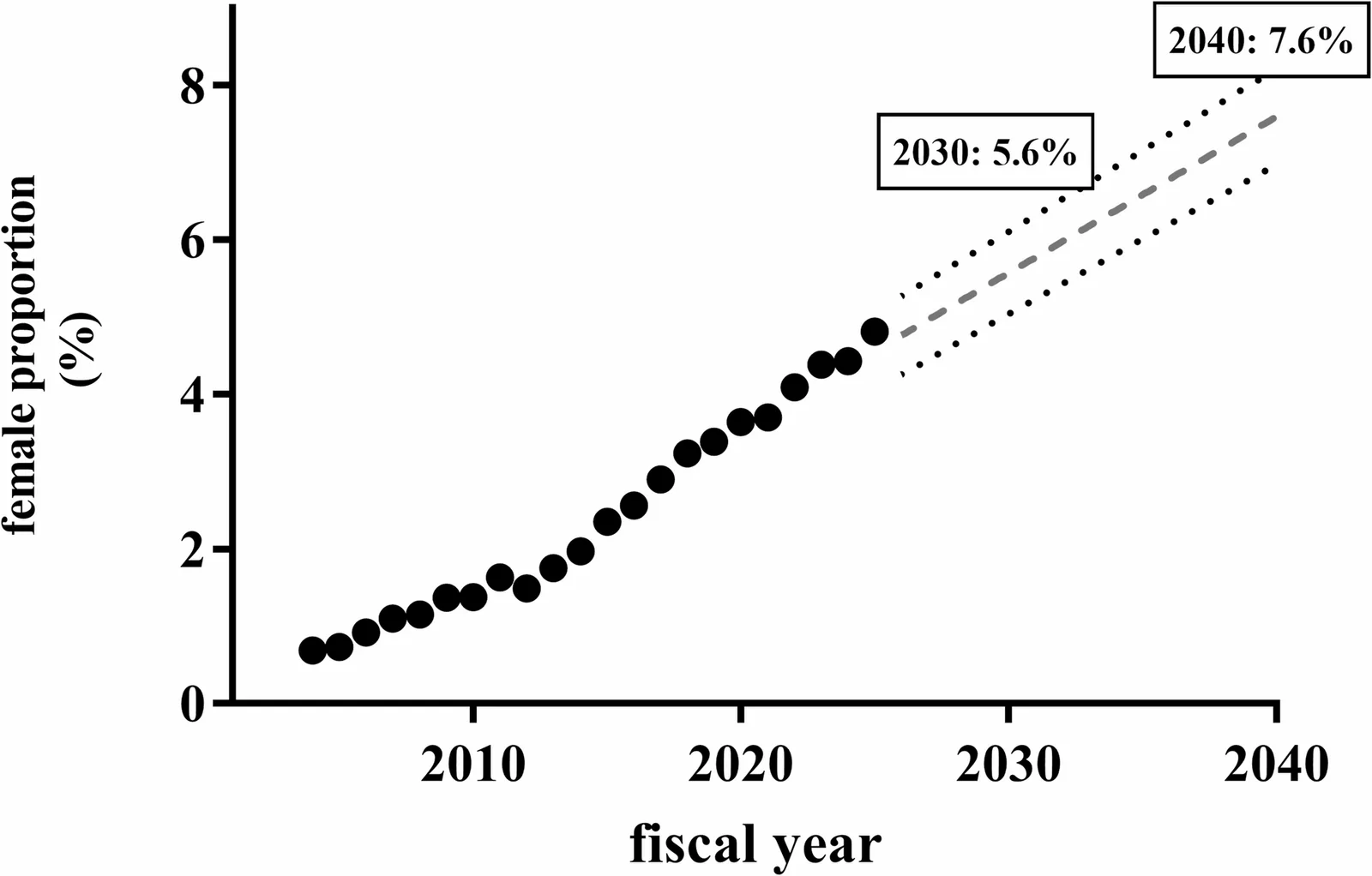
Proportion of Female Board-Certified Cardiovascular Surgeons in Japan, 2004–2025, with Linear Regression and Prediction Intervals Extended to 2040

From 2004 to 2025, the total number of board-certified cardiovascular surgeons in Japan increased from 1453 to 2578 (Table 1). The number of female surgeons rose from 10 (0.7%) in 2004 to 124 (4.8%) in 2025 (Table 1). Linear regression for the absolute number of female surgeons showed an average annual increase of 5.45 surgeons (95% confidence interval [CI] 5.12–5.78; R^2^ = 0.977; *P* < 0.001), with a projection of 195 female surgeons by 2040 (95% prediction interval [PI] 181–209) (Fig. 1). Linear regression analysis revealed an average annual increase of 0.202 percentage points (95% CI 0.18–0.23; R^2^ = 0.973; *P* < 0.001). Extrapolation projected the female proportion to reach 5.6% (95% PI 5.0–6.1%) in 2030, 6.6% (95% PI 6.0–7.2%) in 2035, and 7.6% (95% PI 7.0–8.2%) in 2040 (Fig. 2).

**Table 1:** Number and Proportion of Female Board-Certified Cardiovascular Surgeons in Japan, 2004–2025

Year	Total certified surgeons	Female surgeon	Female proportion (%)	New certifications	New female certifications	New female proportion (%)
2004	1453	10	0.7	39	0	0.0
2005	1643	12	0.7	53	1	1.9
2006	1848	17	0.9	79	4	5.1
2007	1911	21	1.1	37	4	10.8
2008	2002	23	1.1	46	2	4.3
2009	1605	22	1.4	85	3	3.5
2010	1669	23	1.4	100	3	3.0
2011	1716	28	1.6	114	4	3.5
2012	1814	27	1.5	114	3	2.6
2013	1881	33	1.8	91	3	3.3
2014	1979	39	2.0	113	7	6.2
2015	1872	44	2.4	130	5	3.8
2016	1950	50	2.6	140	8	5.7
2017	2070	60	2.9	141	11	7.8
2018	2196	71	3.2	156	11	7.1
2019	2214	75	3.4	117	5	4.3
2020	2280	83	3.6	111	8	7.2
2021	2378	88	3.7	92	5	5.4
2022	2446	100	4.1	117	13	11.1
2023	2557	112	4.4	143	15	10.5
2024	2619	116	4.4	113	8	7.1
2025	2578	124	4.8	145	13	9.0

### New certifications

Among newly certified surgeons, the female proportion showed substantial variability, ranging from 0 to 11.1%, and reaching 9.0% in 2025 (Table 1). The number of new female certifications fluctuated between 0 and 15 annually, with linear regression indicating an average annual increase of 0.55 surgeons (95% CI 0.28–0.82; R^2^ = 0.38; *P* < 0.01) and projections of approximately 14.4 in 2030 (95% PI 7.5–21.3) and 19.7 in 2040 (95% PI 12.8–28.6) (Fig. 1).

### Attrition estimates

Net attrition estimates, derived from year-to-year changes in total counts and annual new certifications, varied across years, with several years indicating no net loss (reported as 0.0%) (Supplementary Table 1). There were substantial fluctuations in the number of surgeons in several years (e.g. a decrease in 2009 and 2015), which were likely due to changes in the board certification system, including revisions to training requirements and renewal criteria. Net attrition rates were generally low among female surgeons, with several years indicating no net loss (or a net gain). A comparable pattern was observed among male surgeons, and there was no consistent evidence of higher net attrition for female surgeons than for male surgeons. In the most recent five years (2021–2025), weighted mean net attrition rates were similar between female surgeons and male surgeons (2.61% vs 2.59%), indicating comparable workforce stability by sex over this period.

### International comparison

Historical data for the United States show female representation in cardiothoracic surgery rising from 2.0% in 1999 to 11.5% in 2024 (Table 2). In Japan, the female proportion has also increased over the same period, from 0.7% in 2004 to 4.8% in 2025, though at a slower pace. Japan's current proportion of 4.8% in 2025 is 60% of the global average (8.0%) and 42–47% of the United States (11.4%) and Europe (10.3%) in 2024 (Table 2).

**Table 2:** Proportion of female cardiac surgeons: historical trends in the united states and current international comparison

Year	United states (%)	Europe (%)	Global average (%)	Japan (%)	Reference
1999	2				Shemin et al. [9]
2010	4.6			1.4	Shemin et al. [10]/Present study
2015	8.1			2.4	Olive et al. [5]/Present study
2018	10.3			3.2	Olive et al [5]/Present study
2024–2025*	11.5	10.3	8.0	4.8	Izumi et al. [1]/Present study

Historical data for the United States are from peer-reviewed reports. Global average and Europe data are from the 2024 EACTS global workforce analysis. Japan's 2025 proportion of 4.8% is approximately half the global average and less than half the current US and European levels, indicating a persistent international disparity.

(*) United States: 448 female surgeons out of 3895; Europe 437 female surgeon out of 4244; Global; 1178 female surgeons out of 14,651.

### Visual representations

Figure 1 illustrates the absolute numbers of female surgeons and new female certifications with linear regression lines extended to 2040, highlighting the steady but limited growth in female numbers. Figure 2 shows the female proportion with linear regression and prediction intervals, demonstrating the slow rise and projected future levels. Table 1 provides the complete annual data, and Table 2 summarizes the historical and current international comparison.

## Discussion

The present study provides the first comprehensive analysis of the 21-year trend in female representation among board-certified cardiovascular surgeons in Japan using complete registry data. The proportion of female surgeons increased gradually from 0.7% in 2004 to 4.8% in 2025, corresponding to an average annual rise of 0.202 percentage points. Although this represents a substantial relative increase, the absolute proportion remains low, and linear extrapolation suggests that Japan will not reach 10% until the early 2050s.

The absolute number of female surgeons rose steadily from 10 to 124 over the study period, while the total number of surgeons grew from 1453 to 2578. The proportion of newly certified surgeons showed considerable variability from year to year, reaching 9.0% in 2025. This indicates that recent cohorts are notably more gender-diverse than the overall workforce. This disparity between new certifications and the existing pool may suggest slow turnover in the specialty and the lingering influence of historically male-dominated cohorts [7, 8].

In surgical fields, it is commonly perceived that female physicians experience higher attrition rates due to childbirth and childcare responsibilities, which limits the feminisation of the overall workforce. However, the present analysis challenges this assumption in the context of Japanese cardiovascular surgery. Net attrition estimates revealed no consistent evidence of higher attrition rates among female surgeons compared to male surgeons. In the most recent five years (2021–2025), the weighted mean net attrition rate was similar for both sexes (2.61% for women versus 2.59% for men). This suggests that female retention is at least comparable to, if not better than, that of men in recent cohorts. The slow rise in the proportion of female surgeons in the workforce is therefore more attributable to historical male-dominated cohorts and slower turnover than differential attrition.

International comparison reveals that Japan's current proportion of 4.8% is around 60% of the global average of 8.0%, and 42–47% of the levels reported in the United States (11.5%) and Europe (10.3%) in 2024. Although direct year-on-year comparisons for Europe are limited by the availability of data, these figures further highlight the ongoing international gap. The same report indicates a combined female proportion of 2.9% in East Asia, though country-specific data are limited and may reflect incomplete registration in some nations. Japan's proportion is higher than this regional average but still substantially lower than Western levels, highlighting a persistent international disparity. For international readers, it is worth noting differences in training structure. In the United States, cardiothoracic surgery certification is typically achieved after a 5-year general surgery residency followed by a 2–3-year fellowship. In Japan, the pathway involves longer general surgical training and specialized cardiovascular training. These differences in training duration and system structure may interact with life events such as childbirth and family responsibilities, potentially influencing gender representation, although the specific impact on female proportions requires further investigation in future studies. Beyond these structural differences, several additional factors may contribute to this slow trajectory. The demanding nature of cardiovascular surgery—characterized by prolonged operative hours, frequent emergency call, and the need for continuous skill maintenance—has been repeatedly identified as a major deterrent for female physicians, particularly during child-rearing years. In Japan, the burden of domestic labor and child-rearing responsibilities may disproportionately affect women in surgical fields, potentially contributing to career interruptions or reduced clinical activity. Such socio-institutional and cultural factors may further compound these challenges.

The benefits of increasing female representation in cardiovascular surgery are well documented. Greater gender diversity has been linked to better patient outcomes, improved team performance and more innovative surgical practices [8]. In recognition of these advantages, international societies have introduced structured initiatives to support women in this field. These include dedicated mentorship programs and scholarships, which have led to quicker progress in North America and Europe [7, 8]. Similar efforts may also be warranted in Japan to accelerate progress towards gender equality, given the growing number of women pursuing board certification. In addition, the physician work-style reform implemented in April 2024, which caps overtime hours at 960 h annually (with exemptions for certain specialties including cardiovascular surgery due to the high emergency nature of the field), has been anticipated to reduce burdens such as long working hours and frequent on-call duties. This could potentially support retention and recruitment of female surgeons in the long term. However, given the short timeframe since its implementation, any substantial acceleration of the linear growth projected in our model is unlikely to be reflected in our data ending in March 2025. The reform does not directly address specialty-specific remaining barriers, such as the need for continuous skill maintenance or unpredictable emergency demands. The impact of this reform on cardiovascular surgery should be evaluated in future longitudinal studies.

This study has several limitations. It relies on aggregated registry data, which lacks individual-level information on age, practice setting, and reasons for decertification. The linear regression model assumes that past trends will continue at a constant rate. However, future changes in the gender composition of medical school entrants, career preferences, or the effects of population decline could alter this trajectory in a nonlinear manner. The registry data does not include information on career stage, leadership positions, or instructor-level certification, which limits our ability to assess gender representation in senior roles. The net attrition estimates reflect changes in certification status rather than direct withdrawal from clinical practice, as the registry does not provide reasons for removal (e.g., non-renewal vs. retirement vs. clinical disengagement). This limits interpretation of the estimates as clinical attrition and highlights the need for future studies with more granular data on reasons for leaving the specialty. In addition, the registry does not capture nuances such as reduced surgical volume, transitions to part-time roles, or shifts to non-clinical roles while maintaining the board title, further limiting the interpretation of net attrition as a direct measure of clinical withdrawal or operative involvement. International comparisons are also limited by variations in the methods used to collect data.

In conclusion, the representation of women in Japanese cardiovascular surgery has shown a gradual but consistent increase over the past two decades, reaching 4.8% in 2025. Recent cohorts show increasing female participation, with comparable net attrition rates between women and men in the most recent period. However, women remain substantially under-represented relative to contemporary international benchmarks. Addressing this gap may require further policy and institutional efforts, including measures that reduce barriers related to workplace culture and customary training practices. Such efforts should focus on recruitment, retention, and career progression.

## Supplementary Information

Below is the link to the electronic supplementary material.

Supplementary Material 1.
