# Supplementary material for: Women in Japanese cardiovascular surgery: 21-year trends (2004–2025), current status and future perspectives
Source: Gen Thorac Cardiovasc Surg. 2026 Apr 30;74(10):963–9. doi: 10.1007/s11748-026-02306-7 (PMC13633351; doi:10.1007/s11748-026-02306-7)
Supplement: Supplementary file 1 [file 11748_2026_2306_MOESM1_ESM.docx]

**Supplement table 1.** Estimated Net Attrition for Female and Male Board-Certified Cardiovascular Surgeons in Japan, 2005–2025

| Year | Female surgeon | New female certifications | Female attrition rate (%) | Male surgeon | New male certifications | Male attrition rate (%) |  |
| --- | --- | --- | --- | --- | --- | --- | --- |
|  |  |  |  |  |  |  |  |
| 2004 | 10 | 0 | NA | 1443 | 39 | NA |  |
| 2005 | 12 | 1 | 0.0 | 1631 | 52 | 0.0 |  |
| 2006 | 17 | 4 | 0.0 | 1831 | 75 | 0.0 |  |
| 2007 | 21 | 4 | 0.0 | 1890 | 33 | 0.0 |  |
| 2008 | 23 | 2 | 0.0 | 1979 | 44 | 0.0 |  |
| 2009 | 22 | 3 | 17.4 | 1583 | 82 | 24.2 |  |
| 2010 | 23 | 3 | 9.1 | 1646 | 97 | 2.1 |  |
| 2011 | 28 | 4 | 0.0 | 1688 | 110 | 4.1 |  |
| 2012 | 27 | 3 | 14.3 | 1787 | 111 | 0.7 |  |
| 2013 | 33 | 3 | 0.0 | 1848 | 88 | 1.5 |  |
| 2014 | 39 | 7 | 3.0 | 1940 | 106 | 0.8 |  |
| 2015 | 44 | 5 | 0.0 | 1828 | 125 | 12.2 |  |
| 2016 | 50 | 8 | 4.5 | 1900 | 132 | 3.3 |  |
| 2017 | 60 | 11 | 2.0 | 2010 | 130 | 1.1 |  |
| 2018 | 71 | 11 | 0.0 | 2125 | 145 | 1.5 |  |
| 2019 | 75 | 5 | 1.4 | 2139 | 112 | 4.6 |  |
| 2020 | 83 | 8 | 0.0 | 2197 | 103 | 2.1 |  |
| 2021 | 88 | 5 | 0.0 | 2290 | 87 | 0.0 |  |
| 2022 | 100 | 13 | 1.1 | 2346 | 104 | 2.1 |  |
| 2023 | 112 | 15 | 3.0 | 2445 | 128 | 1.2 |  |
| 2024 | 116 | 8 | 3.6 | 2503 | 105 | 1.9 |  |
| 2025 | 124 | 13 | 4.3 | 2454 | 132 | 7.2 |  |

Legend: The net attrition rate (%) was estimated as [*N_t−1_*+New*_t_*−*N_t_* / *N_t−1_*]×100, where *N_t−1_* and *N_t_* denote the total number of certified surgeons in the previous and current years, respectively, and New*_t_* denotes new certifications in year *t*. When the estimated net attrition was zero or negative (i.e., no net loss or a net gain), the net attrition rate was reported as 0.0%. Positive values indicate net attrition (a net loss of surgeons).
